# Supplementary figures and images for: A high-resolution model of gene expression during Gossypium hirsutum (cotton) fiber development
Source: BMC Genomics. 2025 Mar 6;26:221. doi: 10.1186/s12864-025-11360-z (PMC11884195; doi:10.1186/s12864-025-11360-z)

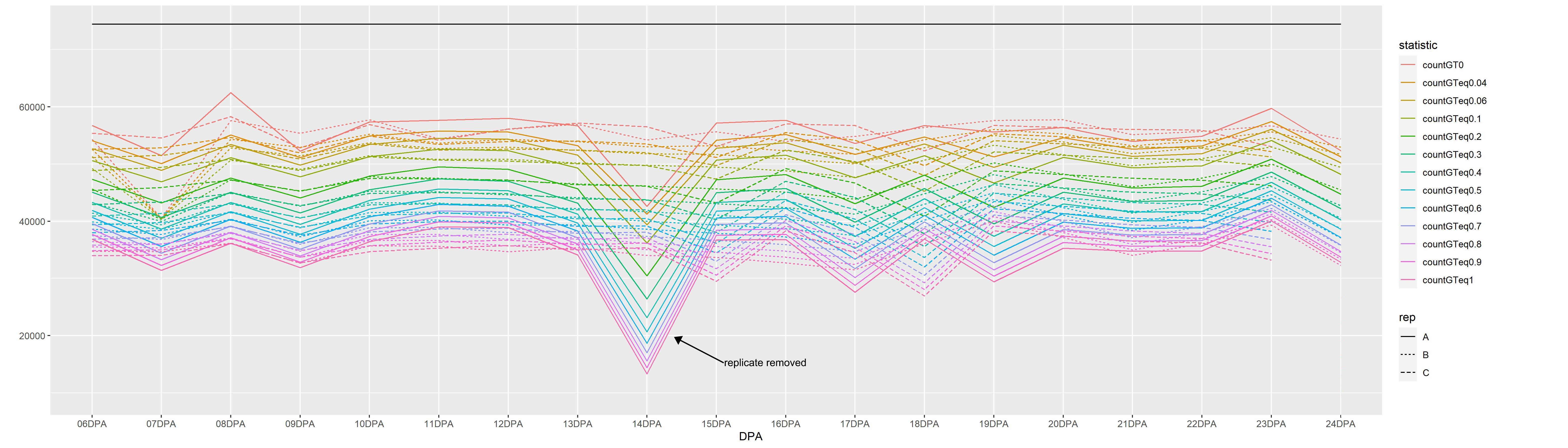

Supplement: Supplementary file 1 — Supplementary Material 1. Figure 1. A graphical representation of the number of genes expressed (y-axis) across developmental time (y-axis). Replicates are noted by linetype. Minimum expression is indicated by line color, where the minimum TPM is determined by the trailing number (e.g., countGT0 indicates TPM > 0, countGTeq1 indicates TPM ≥ 1). [file 12864_2025_11360_MOESM1_ESM.jpg]

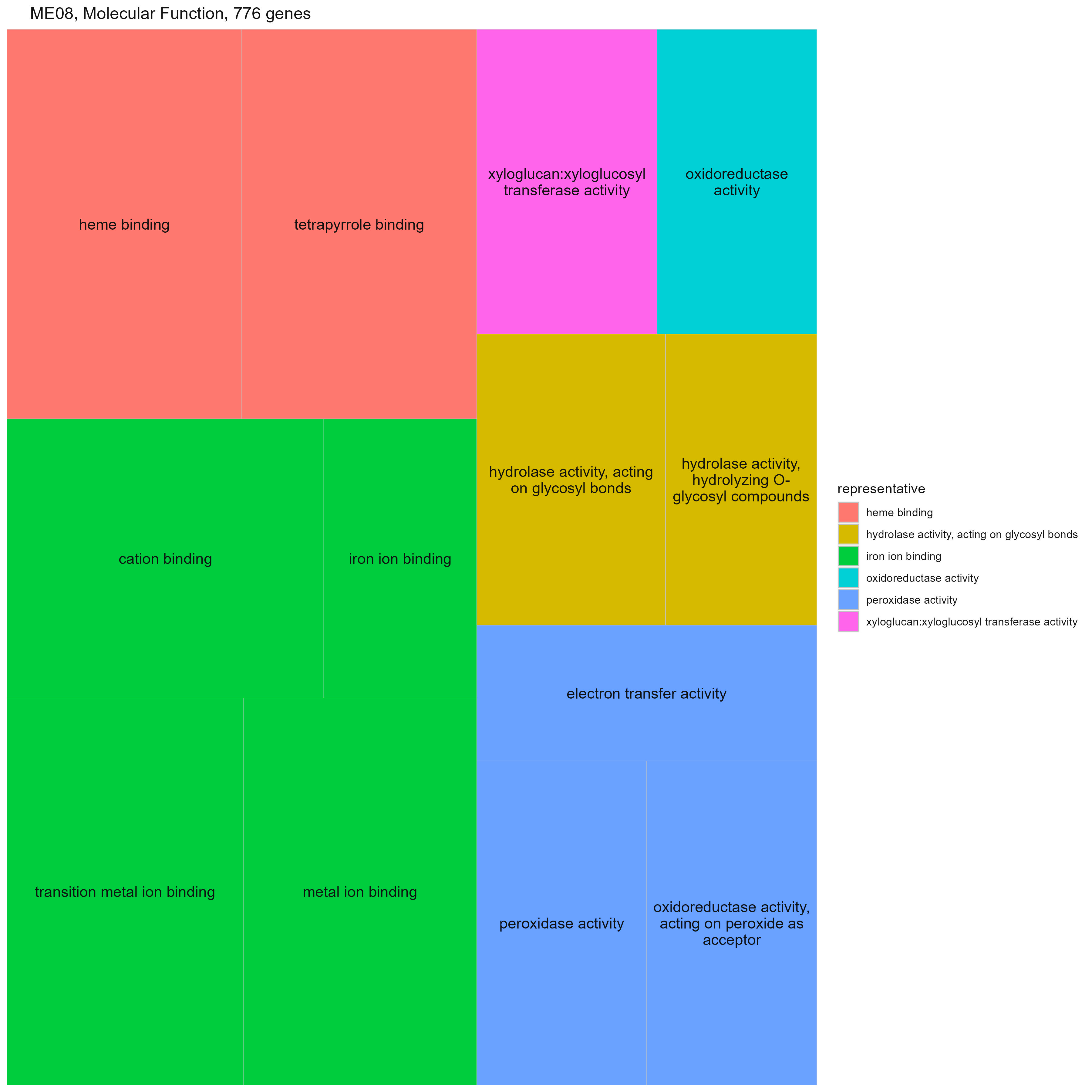

Supplement: Supplementary file 4 — Supplementary Material 4. Figure 4. Molecular function GO enrichment word map for ME8, 776 genes. [file 12864_2025_11360_MOESM4_ESM.jpg]

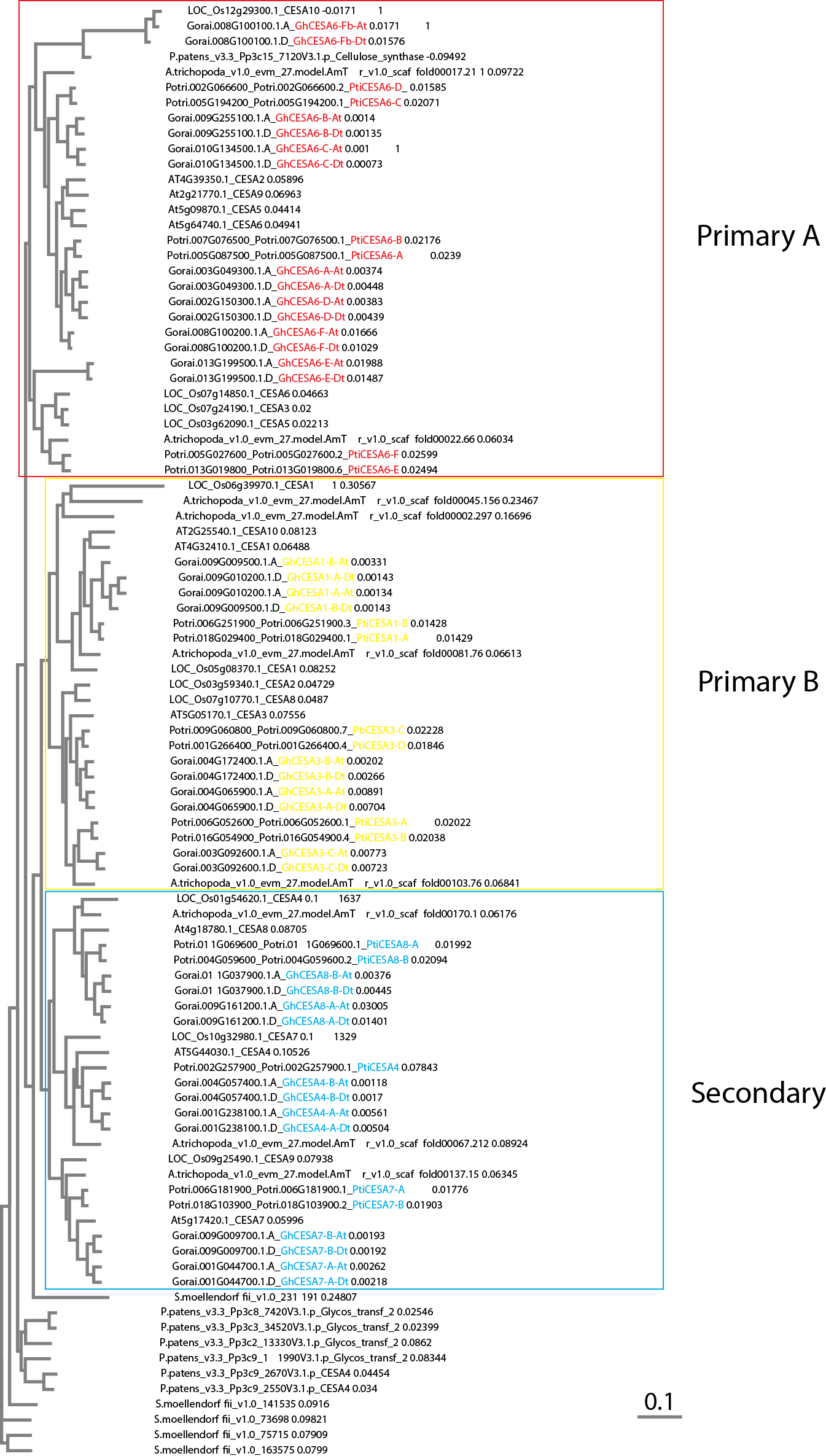

Supplement: Supplementary file 5 — Supplementary Material 5. Figure 5. Phylogenetic analysis of CESA orthologs. CESA protein sequences from Populus trichocarpa [97] and landmark species [98] were downloaded from Phytozome V13 [99] for the analysis. Phylogenetic analysis was performed by Clustal Omega (https://www.ebi.ac.uk/Tools/msa/clustalo/). [file 12864_2025_11360_MOESM5_ESM.jpg]

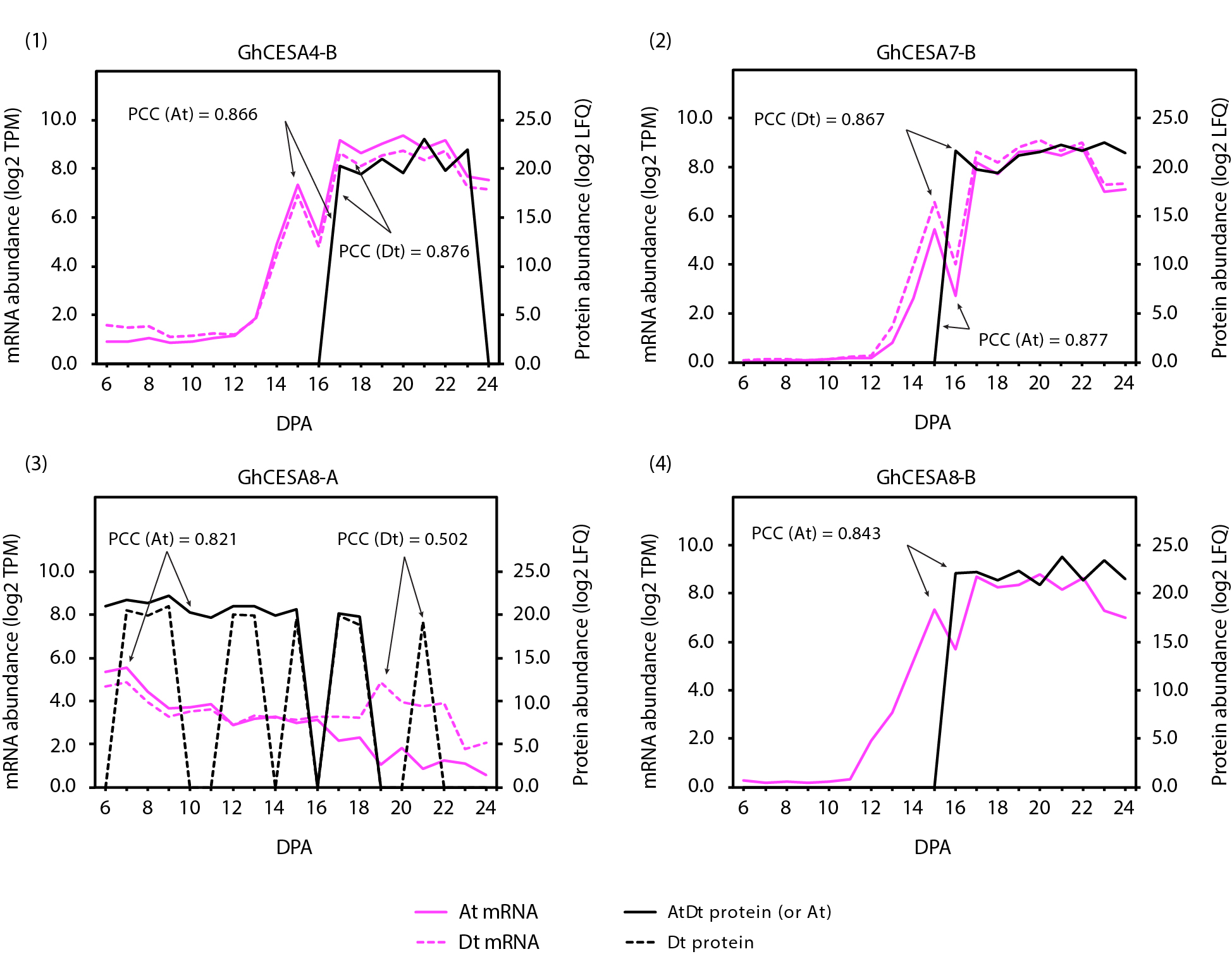

Supplement: Supplementary file 6 — Supplementary Material 6. Figure 6. Profiles of mRNA and protein abundances of selected CESAs that belong to informative groups at protein level. AtDt suffixes reflect ambiguity with respect to homoeolog identification and Dt indicates homoeolog-specific peptides were identified. PCC: Pearson Correlation Coefficient. [file 12864_2025_11360_MOESM6_ESM.jpg]
