# Supplementary material for: A high-resolution model of gene expression during Gossypium hirsutum (cotton) fiber development
Source: BMC Genomics. 2025 Mar 6;26:221. doi: 10.1186/s12864-025-11360-z (PMC11884195; doi:10.1186/s12864-025-11360-z)
Supplement: Supplementary file 3 — Supplementary Material 3. Figure 3. Relative expression of module eigengenes over developmental time. Each module is listed by number and color, as output by WGCNA. The number of genes in each module is listed, and the significance of the module to the developmental timeline (as determined by ANOVA) is listed. [file 12864_2025_11360_MOESM3_ESM.pdf]

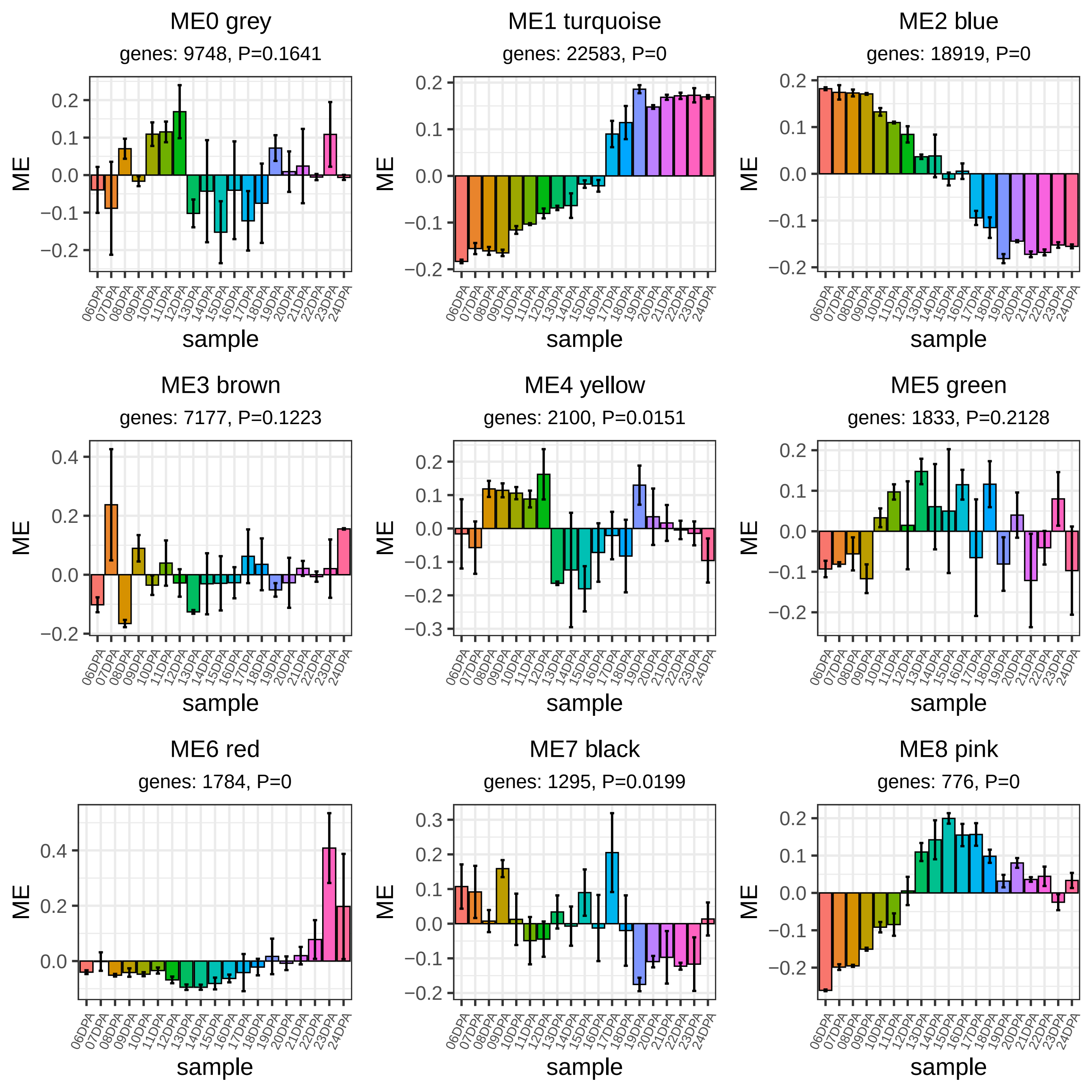
ME0 grey
genes: 9748, P=0.1641
ME1 turquoise
genes: 22583, P=0
ME2 blue
genes: 18919, P=0
ME3 brown
genes: 7177, P=0.1223
ME4 yellow
genes: 2100, P=0.0151
ME5 green
genes: 1833, P=0.2128
ME6 red
genes: 1784, P=0
ME7 black
genes: 1295, P=0.0199
ME8 pink
genes: 776, P=0
ME
sample
06DPA 07DPA 08DPA 09DPA 10DPA 11DPA 12DPA 13DPA 14DPA 15DPA 16DPA 17DPA 18DPA 19DPA 20DPA 21DPA 22DPA 23DPA 24DPA

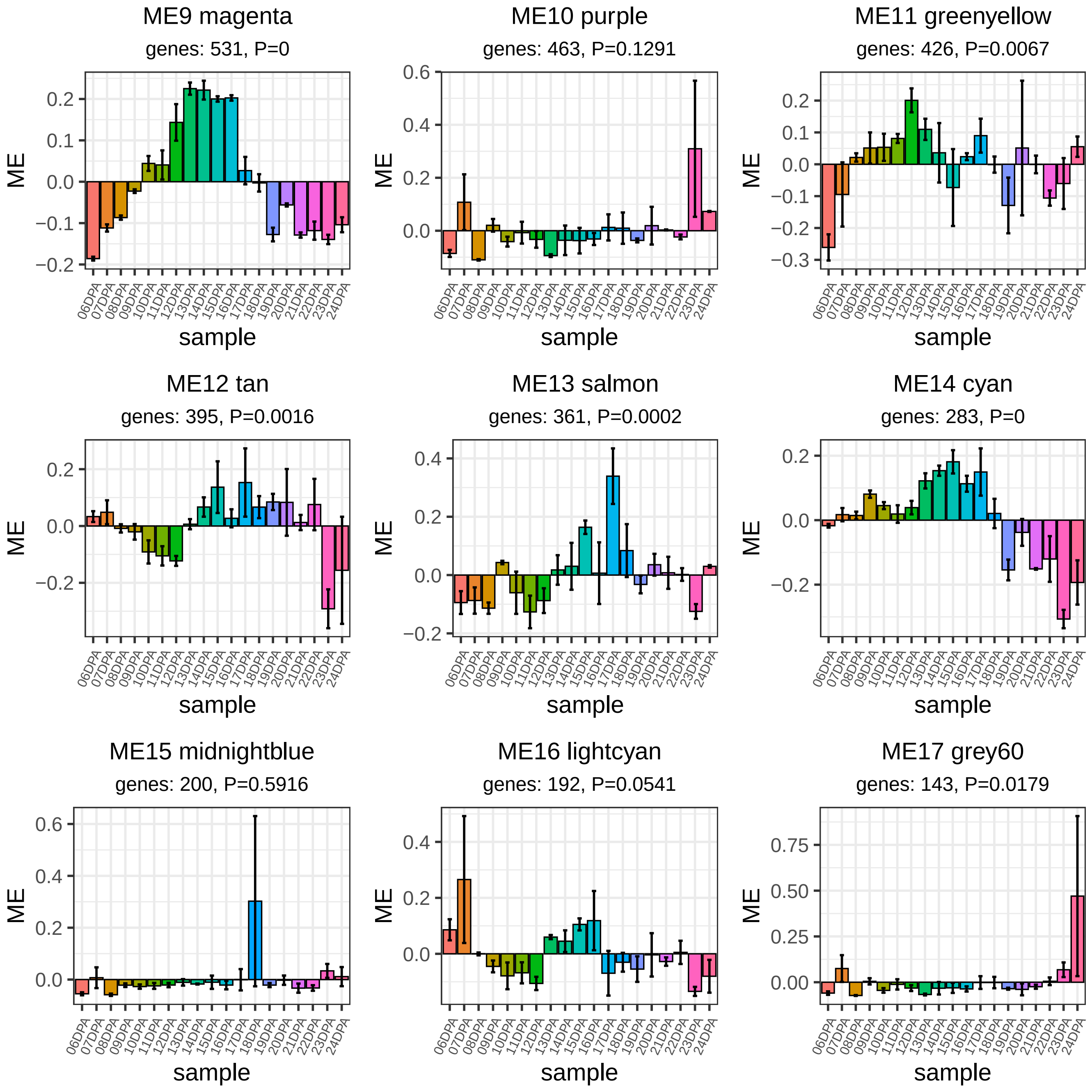
ME9 magenta
genes: 531, P=0
ME10 purple
genes: 463, P=0.1291
ME11 greenyellow
genes: 426, P=0.0067
ME12 tan
genes: 395, P=0.0016
ME13 salmon
genes: 361, P=0.0002
ME14 cyan
genes: 283, P=0
ME15 midnightblue
genes: 200, P=0.5916
ME16 lightcyan
genes: 192, P=0.0541
ME17 grey60
genes: 143, P=0.0179
ME
sample
06DPA 07DPA 08DPA 09DPA 10DPA 11DPA 12DPA 13DPA 14DPA 15DPA 16DPA 17DPA 18DPA 19DPA 20DPA 21DPA 22DPA 23DPA 24DPA
